# Management of the patent ductus arteriosus among infants born at 23 to 32 weeks’ gestation between 2011 to 2022: a report from in the Children’s Hospitals Neonatal Consortium

**DOI:** 10.1038/s41372-025-02257-6

**Published:** 2025-03-15

**Authors:** Mark F. Weems, Molly K. Ball, Isabella Zaniletti, Sharifa Habib, Shannon Hamrick, Theresa R. Grover, Sarah Keene, Karna Murthy, Michael Padula, Ranjit Philip, Rakesh Rao, Shawn Sen, Philip T. Levy, Sharada H. Gowda

**Affiliations:** 1 University of Tennessee Health Science Center and Le Bonheur Children’s Hospital, Memphis, TN USA; 2 The Ohio State University and Nationwide Children’s Hospital, Columbus, OH USA; 3 Children’s Hospitals Neonatal Consortium, Dover, DE USA; 4 University of Toronto and The Hospital for Sick Children, Toronto, ON Canada; 5 Emory University and Children’s Healthcare of Atlanta, Atlanta, GA USA; 6 University of Colorado School of Medicine and Children’s Hospital Colorado, Aurora, CO USA; 7 Northwestern University Feinberg School of Medicine and Ann and Robert H. Lurie Children’s Hospital, Chicago, IL USA; 8 University of Pennsylvania and Children’s Hospital of Philadelphia, Philadelphia, PA USA; 9 Washington University and St. Louis Children’s Hospital, St. Louis, MO USA; 10 Children’s Hospital of Orange County, Orange, CA USA; 11 Harvard Medical School and Boston Children’s Hospital, Boston, MA USA; 12 Baylor College of Medicine and Texas Children’s Hospital, Houston, TX USA

**Keywords:** Paediatrics, Cardiovascular diseases

## Abstract

**Objective:**

This study reports on patent ductus arteriosus (PDA) therapy trends across the Children’s Hospital Neonatal Consortium.

**Study design:**

We performed a 12-year (2011–2022) retrospective study of premature infants (< 33 weeks) with a PDA. We utilized descriptive statistics to compare demographic, inpatient, and discharge characteristics in 3-year epochs.

**Result:**

From 54,813 infants, 19,843 (36%) had a diagnosis of PDA. Use of pharmacotherapy increased 44% (relative) over time, mostly with increased acetaminophen use. There was a 12.7-fold increase in exposure to multiple PDA medications over the study period. While the rate of definitive closure did not change, use of transcatheter PDA closure increased from 0 to 20.3% and surgical ligation decreased from 25.1% to 3.6%.

**Conclusion:**

There has been an increase in the use of multiple pharmacotherapies for PDA, especially among infants born <27 weeks’ gestation. Transcatheter PDA closure has overtaken surgical ligation as the primary method of definitive PDA closure.

## Introduction

A patent ductus arteriosus (PDA) with a resultant shunt volume sufficiently large that cardiovascular compromise may ensue is more common in extremely premature infants [1]. Although uncertainty about how, when, and whether to treat the PDA persist, the strategies aimed at mitigating hemodynamic effects of a PDA include conservative supportive measures, pharmacotherapy, or definitive interventional closure [2]. There have been multiple reports recently addressing shifts in pharmacotherapy for PDA and changes in definitive closure (surgical vs. transcatheter), especially since the introduction of miniaturized transcatheter devices such as the Amplatzer Piccolo™ Occluder (Abbott, Abbott Park, IL, USA) which was approved by the FDA in 2019 [3–9].

While we wait for the results of recent and ongoing clinical trials to fill important gaps in our field’s knowledge, understanding trends in PDA therapies from population-based datasets may help inform the resources needed to provide safe and effective approaches for PDA closure in affected premature infants [10–14]. Accordingly, we investigated PDA management in premature infants born less than 33 weeks’ gestation utilizing a large multicenter neonatal database in Level IV neonatal intensive care units (NICUs). Our study aims to describe changes in management of the PDA in North America from 2011 to 2022 among hospitals participating in the Children’s Hospitals Neonatal Consortium (CHNC) (Dover, DE, USA).

## Materials/subjects and methods

### Study design and patient population

We performed a 12-year (January 2011 through December 2022), retrospective study of prospectively collected data abstracted from the Children’s Hospitals Neonatal Database (CHND). The CHND captures clinical data on infants admitted to 46 participating regional NICUs across North America specializing in referral-based, multi-specialty care for infants, including care for those with a PDA [15]. The methods of data collection and validation in the CHND have been previously published [15]. Briefly, trained abstractors review each patient chart and enter de-identified clinical data into a web-based data collection tool based on standardized clinical definitions. Determination of a PDA diagnosis is made by review of all clinical notes in the patient record.

Infants born from 23 ^0^/_7_ to 32 ^6^/_7_ weeks’ gestation who survived at least 5 days after delivery and were treated in a CHNC NICU from 2011 through 2022 were screened for inclusion and assessed for PDA diagnosis and interventions over the 12-year period. We excluded infants without a PDA diagnosis and patients with incomplete records. We also excluded infants with major congenital anomalies such as complex congenital heart disease, central nervous system anomalies, neuromuscular disorders, facial or airway anomalies, genetic disorders with significant mortality or morbidity, metabolic disorders, skeletal dysplasia, and congenital pulmonary abnormalities.

Patients were stratified into 3-year epochs: (1) 2011–2013, (2) 2014–2016, (3) 2017–2019, and (4) 2020–2022; this interval was chosen to simplify presentation of data in 4 epochs. Demographic, birth data, and clinical characteristics were compared and described over time. PDA treatment was defined as pharmacological (acetaminophen, ibuprofen, or indomethacin) or definitive (surgical ligation or transcatheter device occlusion). Surgical ligation was defined as any surgical technique requiring incision of the chest wall to access the PDA. Transcatheter PDA closure (TCPC) was defined as a procedure utilizing vascular access to achieve endovascular device occlusion of the PDA. Outborn was defined as a patient born at a separate hospital that required transport to the CHNC center. Co-located indicates the patient was born at a hospital connected to the CHNC center, but the initial resuscitation and stabilization occurred at a separate delivery suite/NICU from the CHNC NICU.

We recorded all PDA therapies provided including prior to transfer and after admission to the CHNC NICU. We captured mortality and selected inpatient outcomes, including length of stay and age at discharge. For those patients who stayed at least 30 days in the CHNC center, we collected complications of prematurity at the time of discharge including mild to moderate or severe bronchopulmonary dysplasia (BPD) (following the 2000 NICHD Workshop definition), medical (excluding Bell’s stage 1) and surgical necrotizing enterocolitis (NEC), and retinopathy of prematurity (ROP) Stages 3–5 [16–19].

### Statistical analysis

Summary statistics were used to describe the cohort with PDA by epoch. Continuous variables were reported as median and interquartile range (IQR), and epochs were tested with the non-parametric Kruskal–Wallis Test. Categorical data were reported as count and percentage, and groups were tested with Chi-Square Test. Rates were calculated for each year, and graphical representations were provided by gestational age and intervention. All analyses were performed using SAS Enterprise Guide v8.3 (SAS Institute Inc., Cary, NC, USA). Significance level was evaluated at *p* < 0.05.

## Results

**Fig. 1:**
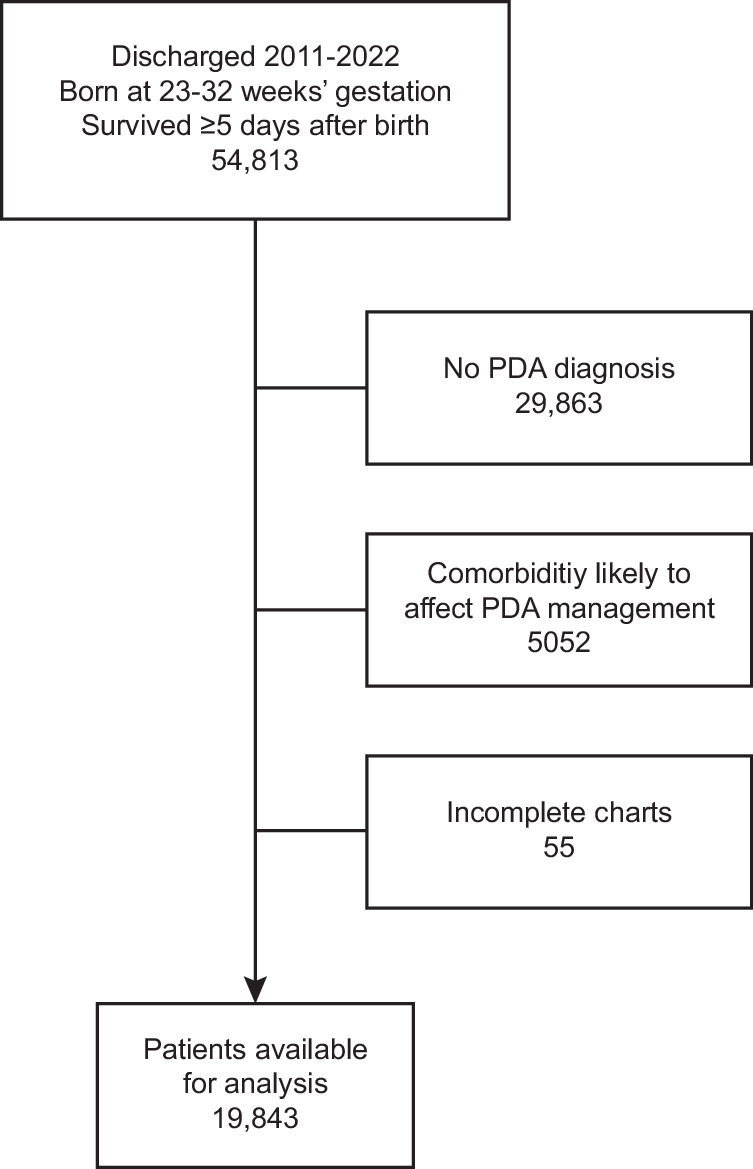
Cohort diagram. *PDA* Patent ductus arteriosus.

From 2011 to 2022, there were 54,813 premature infants cared for at participating centers born from 23 ^0^/_7_ weeks’ to 32 ^6^/_7_ weeks’ gestation. After exclusions, 19,843 (36.2%) of these premature infants with a PDA were analyzed (Fig. 1).

Patient demographics are available in Table 1. Over the study period, we observed increases in participating centers and annual admissions. There was no shift in the distribution of infants by gestational age. The prevalence of small for gestational age (< 10^th^ percentile), frequency of cesarean section, and use of antenatal steroids increased across the epochs while the prevalence of multiple gestation pregnancies decreased.

**Table 1:** Patient demographics.

	Total cohort	(1) 2011–2013	(2) 2014–2016	(3) 2017–2019	(4) 2020–2022	*p*
Total patients, *n*	54,813	10,681	12,894	14,726	16,512	
Patients with PDA diagnosis, *n* (%)	19,843 (36.2)	4051 (37.9)	4732 (36.7)	5257 (35.7)	5803 (35.1)	
CHNC centers, *n*	45	27	32	34	45	
Gestational age, weeks, median (IQR)	26 [24,28]	26 [24,28]	26 [24,28]	26 [24,28]	26 [24,28]	0.397
GA 23–24 weeks, *n* (%)	5911 (29.8)	1126 (27.8)	1425 (30.1)	1588 (30.2)	1772 (30.5)	0.035
GA 25–26 weeks, *n* (%)	6426 (32.4)	1404 (34.7)	1533 (32.4)	1683 (32)	1806 (31.1)	
GA 27–28 weeks, *n* (%)	4000 (20.2)	810 (20)	956 (20.2)	1061 (20.2)	1173 (20.2)	
GA 29–30 weeks, *n* (%)	2032 (10.2)	426 (10.5)	453 (9.6)	528 (10)	625 (10.8)	
GA 31–32 weeks, *n* (%)	1474 (7.4)	285 (7)	365 (7.7)	397 (7.6)	427 (7.4)	
Female, *n* (%)	8931 (45)	1785 (44.1)	2124 (44.9)	2368 (45)	2654 (45.7)	0.430
Male, *n* (%)	10,901 (54.9)	2264 (55.9)	2605 (55.1)	2887 (54.9)	3145 (54.2)	
Birthweight, g, median (IQR)	810 [650,1052]	813 [660,1060]	810 [650,1050]	809 [650,1060]	803 [640,1050]	0.067
<750	8041 (40.5)	1576 (38.9)	1905 (40.3)	2138 (40.7)	2422 (41.7)	0.246
750–999	5909 (29.8)	1264 (31.2)	1423 (30.1)	1538 (29.3)	1684 (29)	
1000–1499	4215 (21.2)	865 (21.4)	1001 (21.2)	1144 (21.8)	1205 (20.8)	
≥1500	1592 (8)	330 (8.1)	377 (8)	408 (7.8)	477 (8.2)	
Birthweight <10^th^ percentile, n (%)	3189 (16.1)	597 (14.7)	693 (14.6)	875 (16.6)	1024 (17.6)	<0.001
Cesarean delivery, *n* (%)	13,827 (69.7)	2757 (68.1)	3236 (68.4)	3654 (69.5)	4180 (72)	<0.001
Antenatal steroids, *n* (%)	12,187 (61.4)	2259 (55.8)	2867 (60.6)	3277 (62.3)	3784 (65.2)	<0.001
Multiple gestation, *n* (%)	4875 (24.6)	1084 (26.8)	1182 (25)	1237 (23.5)	1372 (23.6)	0.001
Age at NICU admission, days, median (IQR)	19 [2,56]	18 [2,50]	18 [2,54]	21 [2,67]	19 [1,53]	<0.001
Inborn, *n* (%)	909 (4.6)	20 (0.5)	57 (1.2)	139 (2.6)	693 (11.9)	.
Born at co-located hospital, *n* (%)	1496 (7.5)	232 (5.7)	446 (9.4)	483 (9.2)	335 (5.8)	.
Outborn, *n* (%)	17,438 (87.9)	3799 (93.8)	4229 (89.4)	4635 (88.2)	4775 (82.3)	<0.001

Data presented as median (interquartile range, IQR) or number (percentage).

*CHNC* Children’s Hospitals Neonatal Consortium, *GA* Gestational age.

The majority of patients (17,438, 87.9%) were outborn and transferred to the CHNC member NICU at a median 24 days of age (IQR 5–64). At admission, these patients had a median postmenstrual age (PMA) of 30 weeks (IQR 27–36) and weight of 1240 g (860–2130). Approximately 12% of these infants were transferred from Level II NICUs and 67% were transferred from Level III NICUs. Transfer from Level IV NICUs was rare (< 1%). PDA diagnosis generally occurred prior to transfer and increased over time; 12,521 (71.8%) had a diagnosis of PDA prior to admission [71.3% in epoch 1 vs. 74.2% in epoch 4 (*p* < 0.001)]. PDA was the primary reason for admission in 2817 (16.2%) of infants, and this increased slightly over time [16.7% in epoch 1 vs. 18.6% in epoch 4 (*p* < 0.001)].

Among outborn patients transferred to a CHNC center, the use of pharmacotherapy for the PDA at the referring hospital varied from 39% to 43.7% across epochs. From epochs 1 to 4, the most common medication used shifted from indomethacin to acetaminophen. The use of multiple medications for PDA increased by 10.8-fold [0.8% in epoch 1 to 8.6% in epoch 4 (*p* < 0.001)] while definitive PDA closure prior to CHNC admission decreased by 59% [6.6% in epoch 1 to 2.7% in epoch 4 (*p* < 0.001)]. PDA-specific therapies given prior to admission to the CHNC center are shown in Table 2.

**Table 2:** Preadmission patent ductus arteriosus therapy among outborn patients.

	Total cohort	(1) 2011–2013	(2) 2014–2016	(3) 2017–2019	(4) 2020–2022	*p*
Patients, *n*	17,438	3799	4229	4635	4775	
Pharmacotherapy for PDA, *n* (%)	7166 (41.1)	1661 (43.7)	1649 (39)	1847 (39.8)	2009 (42.1)	<0.001
Indomethacin only, *n* (%)	4293 (24.6)	1511 (39.8)	1252 (29.6)	958 (20.7)	572 (12)	<0.001
Ibuprofen only, *n* (%)	1196 (6.9)	120 (3.2)	326 (7.7)	364 (7.9)	386 (8.1)	<0.001
Acetaminophen only, *n* (%)	905 (5.2)	0 (0)	2 (0)	261 (5.6)	642 (13.4)	<0.001
Multiple medications for PDA, *n* (%)	772 (4.4)	30 (0.8)	69 (1.6)	264 (5.7)	409 (8.6)	<0.001
Definitive PDA closure, *n* (%)	782 (4.5)	249 (6.6)	220 (5.2)	182 (3.9)	131 (2.7)	<0.001

Data presented as number (percentage).

*PDA* patent ductus arteriosus.

While admitted to the CHNC center, pharmacotherapy for PDA increased 44% over time [14.2% in epoch 1 to 20.4% in epoch 4 (*p* < 0.001)] with a shift from indomethacin to acetaminophen. There was a 12.7-fold increase in the use of multiple medications [0.3% in epoch 1 to 3.8% in epoch 4 (*p* < 0.001)]. PDA-specific therapies given at the CHNC center are shown in Table 3.

**Table 3:** Patent ductus arteriosus therapy in the Children’s Hospitals Neonatal Consortium center.

	Total cohort	(1) 2011–2013	(2) 2014–2016	(3) 2017–2019	(4) 2020–2022	*p*
Patients, *n*	19,843	4051	4732	5257	5803	
Pharmacotherapy for PDA, *n* (%)	3416 (17.2)	577 (14.2)	758 (16)	897 (17.1)	1184 (20.4)	<0.001
Indomethacin only, *n* (%)	1670 (8.4)	525 (13)	524 (11.1)	372 (7.1)	249 (4.3)	<0.001
Ibuprofen only, *n* (%)	553 (2.8)	40 (1)	204 (4.3)	118 (2.2)	191 (3.3)	<0.001
Acetaminophen only, *n* (%)	825 (4.2)	0 (0)	5 (0.1)	298 (5.7)	522 (9)	<0.001
Multiple medications for PDA, *n* (%)	368 (1.9)	12 (0.3)	25 (0.5)	109 (2.1)	222 (3.8)	<0.001
No pharmacotherapy for PDA, *n* (%)	16,427 (87.8)	3474 (85.8)	3974 (84)	4360 (82.9)	4619 (79.6)	<0.001
Definitive PDA closure, *n* (%)	4660 (23.5)	1016 (25.1)	1079 (22.8)	1175 (22.4)	1390 (24)	.
Surgical ligation, *n* (%)	2954 (14.9)	1016 (25.1)	1071 (22.6)	656 (12.5)	211 (3.6)	<0.001
Transcatheter PDA closure, *n* (%)	1706 (8.6)	0 (0)	8 (0.2)	519 (9.9)	1179 (20.3)	<0.001
Age at definitive closure, days, median [IQR]	35 [24,53]	27 [19,38]	30 [21,45]	40 [28,63]	42 [30,62]	<0.001
PMA at definitive closure, weeks, median [IQR]	30.6 [28.7,34]	29.3 [27.7,31.4]	30 [28,32.4]	31.4 [29.1,35.9]	31.9 [29.6,35.6]	<0.001
Weight at definitive closure, g, median [IQR]	1245 [960,1877.5]	1095 [850,1365]	1100 [868,1492.5]	1280 [970,2070]	1327.5 [1020,2025]	<0.001
PDA therapy (pharmacotherapy or definitive closure), *n* (%)	7207 (36.3)	1472 (36.3)	1668 (35.2)	1816 (34.5)	2251 (38.8)	<0.001
No PDA therapy, *n* (%)	12,636 (63.7)	2579 (63.7)	3064 (64.8)	3441 (65.5)	3552 (61.2)	<0.001

Data presented as median (interquartile range, IQR) or number (percentage).

*PDA* patent ductus arteriosus, *PMA* postmenstrual age.

**Fig. 2:**
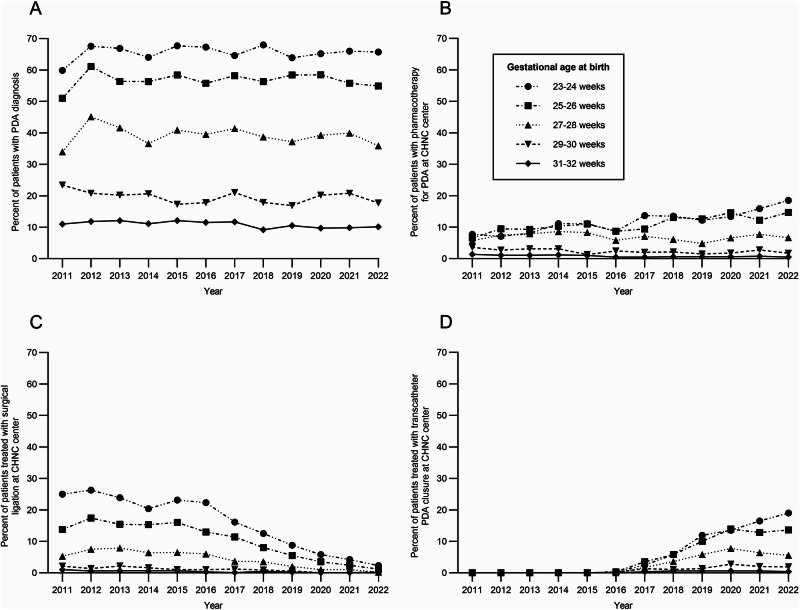
Trends in patent ductus arteriosus (PDA) management in the Children’s Hospital Neonatal Consortium (CHNC). Percent of infants born at 23-32 weeks’ gestation with (**A**) PDA diagnosis in the CHNC center, **B** pharmacotherapy (any) for PDA in the CHNC center, **C** surgical ligation, and **D** transcatheter closure. *n* = 54,813 for all panels, stratified by gestational age at birth.

Over 12 years, rates of definitive closure were similar. Thirty-eight centers performed surgical ligation in 2954 (14.9%) patients with PDA, and 40 centers performed TCPC in 1706 (8.6%) patients during the study period. There was a significant shift in the predominant approach to definitive closure form surgical ligation to TCPC. Surgical ligation decreased from 100% of definitive closures in epoch 1 to 15.2% in epoch 4 (*p* < 0.001). Conversely, TCPC increased from 0 cases in epoch 1 to 84.8% of definitive closures in epoch 4 (*p* < 0.001). The number of centers performing TCPC increased over time, especially since 2019. No centers performed TCPC in epoch 1, but this increased to 39 centers in epoch 4. The panels in Fig. 2 illustrate these trends in (A) PDA diagnosis, (B) pharmacotherapy, (C) surgical ligation, and (D) TCPC. As expected, the diagnosis of PDA was inversely related to gestational age (Fig. 2A). Infants born at 23 to 26 weeks’ gestation had an increase in the use of pharmacotherapy (Fig. 2B). There was a decrease in the use of surgical ligation beginning in 2012 (Fig. 2C) and an increase in TCPC beginning in 2016 (Fig. 2D).

Inpatient disposition data stratified by PDA management are shown in Table 4. In-hospital mortality was highest among those receiving no treatments for PDA while at the CHNC center. Inter-facility transfer occurred most frequently among those receiving TCPC. Among those discharged home, the PMA at discharge was largely similar regardless of PDA therapy given at the CHNC center. These data are presented by epoch in the supplemental Table s1.

**Table 4:** Disposition by patent ductus arteriosus therapy at the Children’s Hospitals Neonatal Consortium center.

	No PDA therapy	Pharmacotherapy for PDA	Surgical ligation	Transcatheter PDA closure
Patients, *n*	12,636	3416	2954	1692
Disposition				
Died, *n* (%)	1615 (12.8)	364 (10.7)	150 (5.1)	69 (4.1)
To home or foster care, *n* (%)	7998 (63.3)	2538 (74.3)	1452 (49.2)	778 (46)
Hospital stay >1 year, *n* (%)	95 (0.8)	9 (0.3)	10 (0.3)	17 (1)
Transfer to another hospital, *n* (%)	2928 (23.2)	505 (14.8)	1342 (45.4)	828 (48.9)
Length of NICU stay, days, median [IQR]	47 [12, 92]	101 [66,133]	80 [10,127]	42 [7,118]
Age at discharge to home or foster care, days, median [IQR]	96 [53,141]	103 [70,136]	97 [43,141]	97 [51,148.5]
PMA at discharge to home or foster care, weeks, median [IQR]	43 [39.6,48.3]	42.4 [39.6,46]	44.5 [41.3,49.6]	45.1 [42,50.4]

Data presented as median (interquartile range, IQR) or number (percentage); columns are not mutually exclusive.

*PDA* Patent ductus arteriosus, *PMA* Postmenstrual age.

A large subgroup (*n* = 12,642, 63.7%) of preterm infants with a diagnosis of PDA were hospitalized longer than 30 days in the CHNC NICUs (supplemental Table s2). When comparing epoch 1 with epoch 4, we identified changes in the frequency of mild-moderate BPD, severe BPD, medical NEC, surgical NEC, and ROP. Mild to moderate BPD was found in 2535 (20.1%) infants [22.4% in epoch 1 vs 17.7% in epoch 4 (*p* < 0.001)], and severe BPD was diagnosed in 5700 (45.1%) infants [35.4% in epoch 1 vs 50.9% in epoch 4 (*p* < 0.001)]. Medical NEC was diagnosed in 1328 (10.5%) infants [14.6% in epoch 1 vs 8.9% in epoch 4 (*p* < 0.001)], and surgical NEC occurred in 1131 (8.9%) infants [10.9% in epoch 1 vs 8% in epoch 4 (*p* = 0.001)]. ROP Stage 3 to 5 occurred in 2627 (20.8%) infants with no observed change over time. Analysis testing associations of these outcomes with PDA therapies are not available.

## Discussion

The management of the PDA in extremely premature infants at risk for cardiovascular compromise remains a long-standing clinical conundrum in the field of neonatology, but understanding contemporary trends of PDA care is a necessary step to filling in knowledge gaps. In this multicenter, 12-year retrospective observational study of infants managed in CHNC NICUs, we identified a shift in the choice of definitive PDA closure from surgical ligation to TCPC. In addition, there were changes in pharmacotherapy including an overall increase in pharmacotherapy use in the most premature infants, increases in acetaminophen and multi-drug therapy, and a decrease in indomethacin use. These trends from a large population-based study serve to complement existing and future randomized control trials results and influence the approaches to PDA management in these high-risk infants [1, 10, 11, 13, 14].

The rapid adoption of TCPC over the study period in CHNC institutions has been balanced by the steady rates of overall definitive closure. TCPC use has grown, beginning in 2016, to 85% of definitive closures during epoch 4 while surgical ligation use has decreased from 100% of definitive closures in epoch 1 to 15% in epoch 4. In contrast, recent publications of other large data sets show a slower adoption of TCPC among networks with a higher proportion of birthing hospitals. Specifically, the Vermont Oxford Network (VON) reported 36% of definitive closures were by surgical ligation in 2020-2022, and data from the Pediatrix Clinical Data Warehouse show that 50% of closures were by surgical ligation in 2020-2021 [3, 8]. We suspected these differences may be related to barriers for establishing a successful TCPC program as technical success increases and major adverse events are less common in centers with at least ten TCPC each year [6, 20]. Furthermore, centers with access to surgical ligation but not TCPC may prefer on-site surgical ligation over transferring a patient to a center with TCPC services, especially for the more premature infants [3, 8].

The trends for definitive closure over the past decade have varied based on the years examined and the gestational ages of included infants. In our study, overall rates of definitive closure amongst babies with a PDA was the same over the 12-year time from 2011 to 2022, mirroring the results from Leahey et al. and the Vermont Oxford Network database from 2018 to 2022 [8]. This is in contrast to Shaw et al. and the Pediatrix Clinical Data Warehouse with a documented decline in rates of any definitive closure from 2014 to 2021 [8]. Lai et al. showed an initial decline in definitive closure rates from 2016 to 2018 followed by a rise from 2018 through 2021 with the Pediatric Health Information System (PHIS) database, likely reflecting a temporal association with the 2019 FDA approval of a PDA occlusion device [5, 9]. Kaluarachchi et al. explored the Neonatal Research Network and observed a decline in procedural closure rates from 2012 to 2021 with infants born at 26 to 28 weeks’ gestation (reflecting the growth of conservative management approaches for the PDA), but there was no change in infants born 22 to 25 weeks [4].

In our study, PDA diagnosis and treatments were inversely related to gestational age at birth. Nearly 70% of infants born at 23 to 24 weeks’ gestation and approximately 10% of infants born at 31 to 32 weeks’ gestation were diagnosed with PDA. As shown in Fig. 2A, there was no meaningful change over time in any gestational age group in the proportion of infants diagnosed with a PDA. The rate of pharmacotherapy decreased slightly for infants born 29 to 32 weeks but increased over time for infants born at 23 to 26 weeks’ gestation. In contrast, two large studies from the Pediatrix Clinical Data Warehouse with primarily inborn patients born less than 30 weeks’ gestation showed a decrease in PDA diagnosis and pharmacotherapy across all gestational ages from 2006 to 2021 [3, 21]. The increased rates of PDA pharmacotherapy in our population of infants born 23 to 26 weeks’ gestation suggests infants transferred to Level IV NICUs are frequently selected to receive treatment for the PDA and may be treated with multiple PDA therapies. In addition, approximately 40% of patients were treated with pharmacotherapy for the PDA prior to admission to a CHNC center, and there has been a shift from indomethacin to acetaminophen over the study period. There was a slight increase in the use of multiple medications prior to admission, but CHND does not collect data on dosing or the number of courses with the same medication. At CHNC centers, there was a similar decrease in indomethacin use and an increase in acetaminophen use for the PDA.

The increase in acetaminophen use, both at the referring center and at the CHNC center, highlights the desire to close the PDA without the side effects of indomethacin or ibuprofen. Several studies suggest that acetaminophen may be an effective therapy with decreased toxicity [22–24]. Early fears of hepatic toxicity seem to have been unfounded with PDA treatment dosing; however, more recent studies have questioned both efficacy and safety [25–28]. Data from the PDA-TOLERATE trial suggests acetaminophen was not as effective as other therapies, and a randomized trial comparing intravenous dosing of acetaminophen to indomethacin was stopped early because acetaminophen was ineffective at attenuating the PDA [26, 29]. Data from the Neonatal Research Network showed a similar increase in acetaminophen use from 2016 to 2020 but also identified an association between acetaminophen use and increased mortality [30]. Animal data suggest the developing lung may be particularly sensitive to acetaminophen toxicity, a finding that may explain acetaminophen-associated morbidity [31, 32]. Our finding that both acetaminophen and multiple PDA therapies have increased over time may support the decreased efficacy of acetaminophen if physicians are using acetaminophen as first-line therapy and choosing another medication if the ductus remains patient after acetaminophen therapy. We did not collect data on the route of administration (intravenous vs. enteral), but some suggest that the enteral approach may be more efficacious [33]. We suspect that extremely premature infants may receive intravenous acetaminophen in the early neonatal period when hemodynamics are unstable or enteral feeding has not been established. All of these factors highlight the need for further research to fully understand the risks, benefits, timing, and optimal route of administration of acetaminophen therapy for PDA in extremely premature infants.

There were unique trends observed concurrent with the rise of TCPC for definitive closure. The use of multiple medications, older age at definitive closure, and a higher weight at PDA closure were all present with patients undergoing TCPC compared to earlier years with higher rates of surgical ligation. We hypothesize there may be a hesitation among clinicians to proceed with definitive closure despite limited evidence from cohort studies that short-term respiratory outcomes and growth velocity are improved when the procedure is performed prior to 4 weeks of age [34, 35]. The ongoing PIVOTAL randomized trial will provide higher level evidence addressing these outcomes [13]. In the meantime, there are several barriers influencing a clinician to delay closure. First, the culture shift from aggressive PDA treatment to more selective intervention paired with conservative management in many patients has taken hold, without a clear detriment to outcomes [36–38]. Second, although the data thus far on TCPC shows low rates of complications, no large trials show efficacy in preventing the negative outcomes associated with a PDA [6, 9]. Similar to the majority of pharmacotherapy studies, the primary outcome assessed in published TCPC studies is simply successful PDA closure with no clear impact on the clinical outcomes of concern. Third, there are practical concerns such as patient size, transport requirements, and clinician awareness of TCPC. The current FDA-approved device is labeled for infants at least 700 g. Although the device has been successfully used in smaller patients, many centers choose to delay closure until the patient is at least 700 g [6, 9, 39]. Not all Level III NICUs have access to TCPC, and these infants must be transported to centers in which TCPC is available. This has both patient safety and financial implications, but with experience and expertise, transport of extremely premature infants can safely be carried out over long distances [40, 41]. In our study, we identified that patients had a shorter length of stay after TCPC than after surgical ligation. Because infants discharged home after ligation or TCPC were discharged at similar ages, the shorter length of stay after TCPC may be in part due to the widespread practice of transferring a patient back to the birth center soon after TCPC. Our data suggest that availability of TCPC is growing rapidly at referral centers, but a lack of guidelines, long term outcome data, and persistent misconceptions about the procedure may cause some clinicians to be reluctant to refer patients for TCPC [1, 42].

This study has several limitations. There are referral biases as this cohort only includes patients transferred to Level IV NICUs and does not represent the entire population of premature infants. The criteria to diagnose and treat a hemodynamically significant PDA, as well as the treatment methods are varied across centers and are changing over time. Though CHNC’s data measures these trends, these results do not have the granularity to discern individual patient differences. Further, medication dosing, duration of therapy, and timing is not available. For example, these data cannot distinguish a patient who received a single dose of ibuprofen for PDA from a patient who received nine doses over three courses. Likewise, there is inconsistency in the diagnostic criteria for PDA which contributes to the center variability in diagnosis and treatment. As the CHND is a voluntary data acquisition platform, data entry errors and missing data from pre-admission records may impact true results compared to our observations. However, standardized training for data abstracters and precise data definitions should optimize data entry accuracy. Finally, the data set ends at NICU discharge. Data on long-term general, cardiopulmonary, and neurodevelopment outcomes are not available.

## Conclusion

In this 12-year large multicenter study, we identified important trends in PDA management. The rate of PDA diagnosis across CHNC NICUs has been stable over time and is inversely related to gestational age. There was an increase in the intent to treat a PDA in infants born less than 27 weeks’ gestation with increases observed in both pharmacotherapy and TCPC. TCPC has become the dominant method of definitive PDA closure relative to surgical ligations. With exciting clinical trials underway, additional research is necessary to understand how to choose optimal therapy and timing for PDA interventions and how these practice changes affect long-term outcomes.

## Supplementary information

Supplemental Tables

## Data Availability

Due to data use agreements among CHNC hospitals, access to network data is limited. Requests for CHNC data should be directed to the CHNC Data Use Committee (exec@thechnc.org).

## References

[1] Mitra S, Bischoff AR, Sathanandam S, Lakshminrusimha S, McNamara PJ. Procedural closure of the patent ductus arteriosus in preterm infants: a clinical practice guideline. J Perinatol. 2024;44:1402–8. 10.1038/s41372-024-02052-9 38997403

[2] Gowda SH, Philip R, Weems MF. Obstacles to the early diagnosis and management of patent ductus arteriosus. Res Rep. Neonatol. 2024;2024:43–57.

[3] Shah ZS, Clark RH, Patt HA, Backes CH Jr, Tolia VN. Trends in procedural closure of the patent ductus arteriosus among infants born at 22 to 30 weeks’ gestation. J Pediatr. 2023;263:113716. 37659585 10.1016/j.jpeds.2023.113716

[4] Kaluarachchi DC, Rysavy MA, Carper BA, Chock VY, Laughon MM, Backes CH, et al. Secular trends in patent ductus arteriosus management in infants born preterm in the national institute of child health and human development neonatal research network. J Pediatr. 2024;266:113877. 38135028 10.1016/j.jpeds.2023.113877 PMC10922632

[5] Lai KC, Richardson T, Berman D, DeMauro SB, King BC, Lagatta J, et al. Current trends in invasive closure of patent ductus arteriosus in very low birth weight infants in United States Children’s Hospitals, 2016-2021. J Pediatr. 2023;263:113712. 37659587 10.1016/j.jpeds.2023.113712

[6] Bischoff AR, Kennedy KF, Backes CH, Sathanandam S, McNamara PJ. Percutaneous closure of the patent ductus arteriosus in infants </=2 kg: IMPACT Registry insights. Pediatrics 2023;152:e2023061460. 10.1542/peds.2023-061460 37529882

[7] Kuntz MT, Staffa SJ, Graham D, Faraoni D, Levy P, DiNardo J, et al. Trend and outcomes for surgical versus transcatheter patent ductus arteriosus closure in neonates and infants at US Children’s Hospitals. J Am Heart Assoc. 2022;11:e022776. 34970919 10.1161/JAHA.121.022776 PMC9075185

[8] Leahy BF, Edwards EM, Ehret DEY, Soll RF, Yeager SB, Flyer JN. Transcatheter and surgical ductus arteriosus closure in very low birth weight infants: 2018-2022. *Pediatrics* 2024;154:e2024065905. 10.1542/peds.2024-065905 39005106

[9] Sathanandam SK, Gutfinger D, O’Brien L, Forbes TJ, Gillespie MJ, Berman DP, et al. Amplatzer Piccolo Occluder clinical trial for percutaneous closure of the patent ductus arteriosus in patients >/=700 grams. Catheter Cardiovasc Inter. 2020;96:1266–76. 10.1002/ccd.28973 PMC7754477 32433821

[10] Gupta S, Subhedar NV, Bell JL, Field D, Bowler U, Hutchison E, et al. Trial of selective early treatment of patent ductus arteriosus with ibuprofen. N. Engl J Med. 2024;390:314–25. 38265644 10.1056/NEJMoa2305582 PMC7615774

[11] Hundscheid T, Onland W, Kooi EMW, Vijlbrief DC, de Vries WB, Dijkman KP, et al. Expectant management or early ibuprofen for patent ductus arteriosus. N. Engl J Med. 2023;388:980–90. 36477458 10.1056/NEJMoa2207418

[12] Mitra S, Hebert A, Castaldo M, Disher T, El-Naggar W, Dhillon S, et al. Selective early medical treatment of the patent ductus arteriosus in extremely low gestational age infants: a pilot randomised controlled trial protocol (SMART-PDA). BMJ Open. 2024;14:e087998. 39053961 10.1136/bmjopen-2024-087998 PMC11284877

[13] Backes C. Preliminary Percutaneous Intervention Versus Observational Trial of Arterial Ductus in Low-weight Infants (PIVOTAL). ClinicalTrials.gov identifier: NCT05547165. 2023 [cited November 21, 2024]Available from: https://clinicaltrials.gov/study/NCT05547165.

[14] Laughon M. Management of the PDA Trial (PDA). ClinicalTrials.gov indentifier: NCT03456336. 2018 [cited November 21, 2024]Available from: https://clinicaltrials.gov/study/NCT03456336.

[15] Murthy K, Dykes FD, Padula MA, Pallotto EK, Reber KM, Durand DJ, et al. The Children’s Hospitals Neonatal Database: an overview of patient complexity, outcomes and variation in care. J Perinatol. 2014;34:582–6. 24603454 10.1038/jp.2014.26

[16] Jobe AH, Bancalari E. Bronchopulmonary dysplasia. Am J Respir Crit Care Med. 2001;163:1723–9. 11401896 10.1164/ajrccm.163.7.2011060

[17] Ehrenkranz RA, Walsh MC, Vohr BR, Jobe AH, Wright LL, Fanaroff AA, et al. Validation of the National Institutes of Health consensus definition of bronchopulmonary dysplasia. Pediatrics. 2005;116:1353–60. 16322158 10.1542/peds.2005-0249

[18] Walsh MC, Kliegman RM. Necrotizing enterocolitis: treatment based on staging criteria. Pediatr Clin North Am. 1986;33:179–201. 3081865 10.1016/S0031-3955(16)34975-6 PMC7131118

[19] International Committee for the Classification of Retinopathy of P. The International Classification of Retinopathy of Prematurity revisited. Arch Ophthalmol. 2005;123:991–9. 16009843 10.1001/archopht.123.7.991

[20] Bischoff AR, Jasani B, Sathanandam SK, Backes C, Weisz DE, McNamara PJ. Percutaneous closure of patent ductus arteriosus in infants 1.5 kg or less: a meta-analysis. J Pediatr. 2021;230:84–92.e14. 33098843 10.1016/j.jpeds.2020.10.035

[21] Bixler GM, Powers GC, Clark RH, Walker MW, Tolia VN. Changes in the diagnosis and management of patent ductus arteriosus from 2006 to 2015 in United States Neonatal Intensive Care Units. J Pediatr. 2017;189:105–12. 28600155 10.1016/j.jpeds.2017.05.024

[22] Hammerman C, Bin-Nun A, Markovitch E, Schimmel MS, Kaplan M, Fink D. Ductal closure with paracetamol: a surprising new approach to patent ductus arteriosus treatment. Pediatrics. 2011;128:e1618–1621. 22065264 10.1542/peds.2011-0359

[23] Terrin G, Conte F, Oncel MY, Scipione A, McNamara PJ, Simons S, et al. Paracetamol for the treatment of patent ductus arteriosus in preterm neonates: a systematic review and meta-analysis. Arch Dis Child Fetal Neonatal Ed. 2016;101:F127–136. 26283668 10.1136/archdischild-2014-307312

[24] Mitra S, Florez ID, Tamayo ME, Mbuagbaw L, Vanniyasingam T, Veroniki AA, et al. Association of placebo, indomethacin, ibuprofen, and acetaminophen with closure of hemodynamically significant patent ductus arteriosus in preterm infants: a systematic review and meta-analysis. JAMA. 2018;319:1221–38. 29584842 10.1001/jama.2018.1896 PMC5885871

[25] Bahrami R, Ezzatabadi A, Mehdizadegan N, Mohammadi H, Amoozgar H, Edraki M. Does high dose intravenous acetaminophen affect liver function for PDA closure in premature neonate? Ital J Pediatr. 2021;47:37. 33596978 10.1186/s13052-020-00940-2 PMC7890839

[26] Davidson JM, Ferguson J, Ivey E, Philip R, Weems MF, Talati AJ. A randomized trial of intravenous acetaminophen versus indomethacin for treatment of hemodynamically significant PDAs in VLBW infants. J Perinatol. 2021;41:93–99. 32439957 10.1038/s41372-020-0694-1

[27] Sridharan K, Al Jufairi M, Al Ansari E, Al Marzooq R, Hubail Z, Hasan SJR, et al. Intravenous acetaminophen (at 15 mg/kg/dose every 6 h) in critically ill preterm neonates with patent ductus arteriosus: A prospective study. J Clin Pharm Ther. 2021;46:1010–9. 33638909 10.1111/jcpt.13384

[28] Wright CJ, McCulley DJ, Mitra S, Jensen EA. Acetaminophen for the patent ductus arteriosus: has safety been adequately demonstrated? J Perinatol. 2023;43:1230–7. 37169914 10.1038/s41372-023-01697-2 PMC10626600

[29] Liebowitz M, Kaempf J, Erdeve O, Bulbul A, Hakansson S, Lindqvist J, et al. Comparative effectiveness of drugs used to constrict the patent ductus arteriosus: a secondary analysis of the PDA-TOLERATE trial (NCT01958320). J Perinatol. 2019;39:599–607. 30850756 10.1038/s41372-019-0347-4 PMC6561645

[30] Jensen EA, DeMauro SB, Rysavy MA, Patel RM, Laughon MM, Eichenwald EC, et al. Acetaminophen for patent ductus arteriosus and risk of mortality and pulmonary morbidity. Pediatrics 2024;154:e2023065056. 10.1542/peds.2023-065056 PMC11291959 39011550

[31] Dobrinskikh E, Al-Juboori SI, Zarate MA, Zheng L, De Dios R, Balasubramaniyan D, et al. Pulmonary implications of acetaminophen exposures independent of hepatic toxicity. Am J Physiol Lung Cell Mol Physiol. 2021;321:L941–L953. 34585971 10.1152/ajplung.00234.2021 PMC8616618

[32] Dobrinskikh E, Sherlock LG, Orlicky DJ, Zheng L, De Dios R, Balasubramaniyan D, et al. The developing murine lung is susceptible to acetaminophen toxicity. Am J Physiol Lung Cell Mol Physiol. 2021;320:L969–L978. 33759579 10.1152/ajplung.00072.2021 PMC8174833

[33] Gover A, Levy PT, Rotschild A, Golzman M, Molad M, Lavie-Nevo K, et al. Oral versus intravenous paracetamol for patent ductus arteriosus closure in preterm infants. Pediatr Res. 2022;92:1146–52. 35087197 10.1038/s41390-022-01944-w

[34] Philip R, Waller BR, Chilakala S, Graham B, Stecchi N, Apalodimas L, et al. Hemodynamic and clinical consequences of early versus delayed closure of patent ductus arteriosus in extremely low birth weight infants. J Perinatol. 2021;41:100–8. 32792636 10.1038/s41372-020-00772-2

[35] Sathanandam S, Balduf K, Chilakala S, Washington K, Allen K, Knott-Craig C, et al. Role of Transcatheter patent ductus arteriosus closure in extremely low birth weight infants. Catheter Cardiovasc Inter. 2019;93:89–96. 10.1002/ccd.27808 30269408

[36] Altit G, Saeed S, Beltempo M, Claveau M, Lapointe A, Basso O. Outcomes of extremely premature infants comparing patent ductus arteriosus management approaches. J Pediatr. 2021;235:49–57.e42. 33864797 10.1016/j.jpeds.2021.04.014

[37] Sung SI, Chang YS, Chun JY, Yoon SA, Yoo HS, Ahn SY, et al. Mandatory closure versus nonintervention for patent ductus arteriosus in very preterm infants. J Pediatr. 2016;177:66–71.e61. 27453374 10.1016/j.jpeds.2016.06.046

[38] Sung SI, Lee MH, Ahn SY, Chang YS, Park WS. Effect of nonintervention vs oral ibuprofen in patent ductus arteriosus in preterm infants: a randomized clinical trial. JAMA Pediatr 2020;174**:**755–63. 10.1001/jamapediatrics.2020.1447 PMC7296457 32539121

[39] Fernandez MC, Kase JS, Giamelli J, Reichlin A. Morbidity and neurodevelopmental outcomes at 2 years in preterm infants undergoing percutaneous transcatheter closure vs. surgical ligation of the PDA. J Perinatol. 2024;44:1454–62. 10.1038/s41372-024-02019-w 38831120

[40] Aw TC, Chan B, Singh Y. Transport and anaesthesia consideration for transcatheter patent ductus arteriosus closure in premature infants. J Cardiovasc Dev Dis 2023;10:377. 10.3390/jcdd10090377 PMC10531776 37754806

[41] Willis A, Pereiras L, Head T, Dupuis G, Sessums J, Corder G, et al. Transport of extremely low birth weight neonates for persistent ductus arteriosus closure in the catheterization lab. Congenit Heart Dis. 2019;14:69–73. 30811788 10.1111/chd.12706

[42] Sathanandam S, Whiting S, Cunningham J, Zurakowski D, Apalodimas L, Waller BR, et al. Practice variation in the management of patent ductus arteriosus in extremely low birth weight infants in the United States: Survey results among cardiologists and neonatologists. Congenit Heart Dis. 2019;14:6–14. 30811803 10.1111/chd.12729

