# Supplementary material for: Management of the patent ductus arteriosus among infants born at 23 to 32 weeks’ gestation between 2011 to 2022: a report from in the Children’s Hospitals Neonatal Consortium
Source: J Perinatol. 2025 Mar 15;45(11):1540–7. doi: 10.1038/s41372-025-02257-6 (PMC12660138; doi:10.1038/s41372-025-02257-6)
Supplement: Supplementary file 1 — Supplemental Tables [file 41372_2025_2257_MOESM1_ESM.pdf]

Management of the Patent Ductus Arteriosus among Infants Born at 23 to 32 Weeks' Gestation between 2011 to 2022: A Report from in the Children's Hospitals Neonatal Consortium

Mark F Weems, Molly K Ball, Isabella Zaniletti, Sharifa Habib, Shannon Hamrick, Theresa Grover, Sarah Keene, Karna Murthy, Michael Padula, Ranjit Philip, Rakesh Rao, Shawn Sen, Philip T Levy, Sharada H Gowda, on Behalf of the CHNC Cardiac Focus Group

Supplemental Tables

Table s1. Disposition by PDA therapy at the CHNC hospital. Columns are not mutually exclusive.

	Total cohort	(1) 2011-2013	(2) 2014-2016	(3) 2017-2019	(4) 2020-2022	p
**No PDA Therapy**						
**Patients**	**12,636**	**2579**	**3064**	**3441**	**3552**	
**Disposition**						
**Died, n (%)**	1615 (12.8)	352 (13.6)	396 (12.9)	427 (12.4)	440 (12.4)	<0.001
**To home or foster care, n (%)**	7998 (63.3)	1547 (60)	1965 (64.1)	2148 (62.4)	2338 (65.8)	.
**Hospital stay >1 year, n (%)**	95 (0.8)	14 (0.5)	22 (0.7)	25 (0.7)	34 (1)	.
**Transfer to another hospital, n (%)**	2928 (23.2)	666 (25.8)	681 (22.2)	841 (24.4)	740 (20.8)	.
**Length of NICU stay, days, median [IQR]**	47 [12,92]	39 [10,83]	46 [12,91]	46 [12,91]	52 [14,99.5]	<0.001
**Age at discharge to home or foster care, days, median [IQR]**	96 [53,141]	91 [51,131]	98 [53,144]	98 [57,143]	96 [53,142]	<0.001
**PMA at discharge to home or foster care, weeks, median [IQR]**	43 [39.6,48.3]	42.4 [39.3,47.3]	43.1 [39.7,48.7]	43.4 [39.9,48.6]	43.3 [39.7,48.1]	<0.001
**Pharmacotherapy for PDA**						
**Patients**	**3416**	**577**	**758**	**897**	**1184**	
**Disposition**						
**Died, n (%)**	364 (10.7)	55 (9.5)	67 (8.8)	106 (11.8)	136 (11.5)	0.19
**To home or foster care, n (%)**	2538 (74.3)	429 (74.4)	583 (76.9)	644 (71.8)	882 (74.5)	.
**Hospital stay >1 year, n (%)**	9 (0.3)	0 (0)	1 (0.1)	4 (0.4)	4 (0.3)	.

Transfer to another hospital, n (%)	505 (14.8)	93 (16.1)	107 (14.1)	143 (15.9)	162 (13.7)	.
**Length of NICU stay, days, median [IQR]**	101 [66,133]	88 [60,118]	94 [64,123]	103 [68,137]	110 [73,142]	<0.001
**Age at discharge to home or foster care, days, median [IQR]**	103 [70,136]	90 [60,119]	97 [68,126]	109 [74,142]	113 [79,148]	<0.001
**PMA at discharge to home or foster care, weeks, median [IQR]**	42 [40,46]	41 [38,44]	41 [39,44]	43 [40,47]	44 [40,48]	<0.001
**Surgical Ligation**						
**Patients**	**2954**	**1016**	**1071**	**656**	**211**	
**Disposition**						
**Died, n (%)**	150 (4.9)	50 (4.9)	53 (4.9)	33 (5)	14 (6.6)	0.054
**To home or foster care, n (%)**	1452 (49.2)	460 (45.3)	547 (51.1)	325 (49.5)	120 (56.9)	.
**Hospital stay >1 year, n (%)**	10 (0.3)	3 (0.3)	3 (0.3)	3 (0.5)	1 (0.5)	.
**Transfer to another hospital, n (%)**	1342 (45.4)	503 (49.5)	468 (43.7)	295 (45)	76 (36)	.
**Length of NICU stay, days, median [IQR]**	80 [10,127]	56 [8,115]	78 [10,124]	92 [11,137]	119 [24,155]	<0.001
**Age at discharge to home or foster care, days, median [IQR]**	97 [43,141]	79 [38,126]	98 [44,138]	106 [51,150]	135 [55,186]	<0.001
**PMA at discharge to home or foster care, weeks, median [IQR]**	45 [41,50]	43 [40,47]	44 [41,50]	45 [43,50]	49 [44,57]	<0.001
**Transcatheter PDA Closure**						
**Patients**	**1692**	**0**	**8**	**516**	**1168**	
**Disposition**						
**Died, n (%)**	69 (4.1)	0 (0)	0 (0)	21 (4.1)	48 (4.1)	0.37
**To home or foster care, n (%)**	778 (46)	0 (0)	5 (62.5)	258 (50)	515 (44.1)	.
**Hospital stay >1 year, n (%)**	17 (1)	0 (0)	0 (0)	4 (0.8)	13 (1.1)	.

**Transfer to another hospital, n (%)**	828 (48.9)	0 (0)	3 (37.5)	233 (45.2)	592 (50.7)	.
**Length of NICU stay, days, median [IQR]**	42 [7,118]	0 (0)	53 [15,89]	39 [7,112]	43 [7,120]	0.87
**Age at discharge to home or foster care, days, median [IQR]**	97 [51,149]	0 (0)	96 [78,148]	101 [57,155]	95 [49,145]	0.11
**PMA at discharge to home or foster care, weeks, median [IQR]**	45 [42,50]	0 (0)	46 [43,46]	46 [41,52]	45 [42,50]	0.91

PDA Patent ductus arteriosus, *CHNC* Children's Hospitals Neonatal Consortium, *PMA* Postmenstrual age.

Table s2. Outcomes of infants with PDA hospitalized longer than 30 days in the CHNC NICU.

	**Total cohort**	**(1)2011-2013**	**(2)2014-2016**	**(3) 2017-2019**	**(4) 2020-2022**	**p**
**Patients, n**	12,642	2414	3090	3359	3779	
**Mild-moderate BPD, n (%)**	2535 (20.1)	540 (22.4)	660 (21.4)	667 (19.9)	668 (17.7)	<0.001
**Severe BPD, n (%)**	5700 (45.1)	855 (35.4)	1257 (40.7)	1666 (49.6)	1922 (50.9)	<0.001
**Medical NEC, n (%)**	1328 (10.5)	352 (14.6)	295 (9.5)	343 (10.2)	338 (8.9)	<0.001
**Surgical NEC, n (%)**	1131 (8.9)	262 (10.9)	255 (8.3)	313 (9.3)	301 (8)	0.001
**ROP Stage 3-5, n (%)**	2627 (20.8)	475 (19.7)	629 (20.4)	735 (21.9)	788 (20.9)	0.203

PDA Patent ductus arteriosus, *CHNC* Children's Hospitals Neonatal Consortium, *NICU* Neonatal intensive care unit, *BPD* Bronchopulmonary dysplasia, *NEC* Necrotizing Enterocolitis, *ROP* Retinopathy of prematurity.
